# Combined Effects of Exercise and Denosumab Treatment on Local Failure in Post-menopausal Osteoporosis–Insights from Bone Remodelling Simulations Accounting for Mineralisation and Damage

**DOI:** 10.3389/fbioe.2021.635056

**Published:** 2021-06-04

**Authors:** Javier Martínez-Reina, José L. Calvo-Gallego, Peter Pivonka

**Affiliations:** ^1^Departamento de Ingeniería Mecánica y Fabricación, Universidad de Sevilla, Seville, Spain; ^2^School of Mechanical, Medical and Process Engineering, Queensland University of Technology, Brisbane, QLD, Australia

**Keywords:** post-menopausal osteoporosis, bone turnover, bone mineralisation, denosumab, PK-PD modelling, bone mineral density, risk of failure, damage

## Abstract

Denosumab has been shown to increase bone mineral density (BMD) and reduce the fracture risk in patients with post-menopausal osteoporosis (PMO). Increase in BMD is linked with an increase in bone matrix mineralisation due to suppression of bone remodelling. However, denosumab anti-resorptive action also leads to an increase in fatigue microdamage, which may ultimately lead to an increased fracture risk. A novel mechanobiological model of bone remodelling was developed to investigate how these counter-acting mechanisms are affected both by exercise and long-term denosumab treatment. This model incorporates Frost's mechanostat feedback, a bone mineralisation algorithm and an evolution law for microdamage accumulation. Mechanical disuse and microdamage were assumed to stimulate RANKL production, which modulates activation frequency of basic multicellular units in bone remodelling. This mechanical feedback mechanism controls removal of excess bone mass and microdamage. Furthermore, a novel measure of bone local failure due to instantaneous overloading was developed. Numerical simulations indicate that trabecular bone volume fraction and bone matrix damage are determined by the respective bone turnover and homeostatic loading conditions. PMO patients treated with the currently WHO-approved dose of denosumab (60 mg administrated every 6 months) exhibit increased BMD, increased bone ash fraction and damage. In untreated patients, BMD will significantly decrease, as will ash fraction; while damage will increase. The model predicted that, depending on the time elapsed between the onset of PMO and the beginning of treatment, BMD slowly converges to the same steady-state value, while damage is low in patients treated soon after the onset of the disease and high in patients having PMO for a longer period. The simulations show that late treatment PMO patients have a significantly higher risk of local failure compared to patients that are treated soon after the onset of the disease. Furthermore, overloading resulted in an increase of BMD, but also in a faster increase of damage, which may consequently promote the risk of fracture, specially in late treatment scenarios. In case of mechanical disuse, the model predicted reduced BMD gains due to denosumab, while no significant change in damage occurred, thus leading to an increased risk of local failure compared to habitual loading.

## 1. Introduction

Denosumab treatment of patients with post-menopausal osteoporosis (PMO) has been shown to increase bone mineral density (BMD) as assessed by dual-energy X-ray absorptiometry (DXA) and it was observed that denosumab reduced the risk of new radiographic vertebral fractures by 68%, with the risk of hip fractures and non-vertebral fractures decreasing by 40 and 20%, respectively (Cummings et al., 2009). The increase in BMD has been attributed to a decrease in bone turnover and the associated increase in the degree of bone mineralisation (Dempster et al., 2018), as the osteoclastic activity dissolves the bone matrix and prevents it from being mineralised further. Despite its high efficacy in the treatment of PMO, long-term (i.e., >4 years) treatment with denosumab has also been linked with certain risks. Among those, development of atypical femoral fractures (AFF) is of significant concern and has been associated with the accumulation of microcracks in the bone matrix due to suppression of bone remodelling (Aspenberg, 2014), which is detected by a decrease in the levels of bone turnover markers (Miller et al., 2008).

Action of anti-resorptive drugs has been extensively studied experimentally and linked to decreases in bone turnover and suppression of osteoclastic activity together with observed changes in BMD (Langdahl, 2020). Riggs and Parfitt (2005) suggested that there are potentially three mechanisms on how anti-resorptive drugs exert increases in BMD: (i) increases of bone mineralisation, (ii) accumulation of microcracks in the bone matrix, and (iii) positive bone balance due to net increase of osteoblasts compared to osteoclasts. The latter mechanism which theoretically could lead to overfilling of resorption cavities in trabecular bone has more recently been ruled out due to the fact that bone remodelling is a coupled process, and suppression of osteoclastic activity also leads to reduction of osteoblastic activity (Sims and Martin, 2015). Hence, understanding on how the first two mechanisms interact and regulate BMD is central to understanding action of anti-resorptive drugs. However, it is currently not known to which extent the two mechanisms of increases of bone mineralisation and accumulation of microcracks contribute to net BMD increases. The aim of the current paper is to add to our understanding of the relative contributions of these competing mechanism which can provide new insights into the efficacy and safety of long-term treatment of OP with denosumab (and other anti-resorptive drugs). In particular, this new knowledge would help design new treatment regimens with respect to drug duration, dose magnitude, and dosing intervals.

Increases in BMD due to anti-resorptive treatments are explained by the conceptual model of bone mineralisation. This model links the rate of bone remodelling (i.e., bone turnover) with the degree of bone tissue mineralisation (BTM) (Bala et al., 2013; Boivin and Meunier, 2002). Bone mineralisation has two phases: a fast primary phase, which takes place over several days to weeks and achieves a degree of mineralisation of approximately 70% and a slow secondary phase, which can take from months to years and may achieve degrees of mineralisation of up to 95%. On the other hand, mineral is removed from the bone matrix by osteoclastic action, which dissolves it, returning it to the bloodstream. In this manner, the model predicts that bone sites undergoing high turnover are characterised by a lower BTM (and BMD) based on the fact that continuous remodelling prevents excessive secondary mineralisation. On the contrary, at sites of low turnover there is sufficient time for secondary mineralisation to occur and the mineral content is quite high, as occurs for example in interstitial bone (O'Brien et al., 2000).

The mechanism of targeted remodelling was first discovered in the late 90s looking at histological sections of normal, healthy cortical bone which showed microcracks around osteons which have been associated with exposure to dynamic habitual loading (e.g., walking) (Burr et al., 1985; Parfitt, 2002). In engineering, these microcracks are commonly referred to as fatigue microcracks, which accumulate in a material exposed to dynamic loading, eventually coalescing into macrocracks and leading to fracture (i.e., structural failure). Targeted bone remodelling is the process by which these microcracks are removed from the bone matrix in order to avoid development of fatigue failure or occurrence of stress fractures. While originally observed mostly in cortical bone, fatigue damage may also occur in trabecular bone (Dendorfer et al., 2006; Rapillard et al., 2006). However, in trabecular bone microcracks cannot easily accumulate due to the fact that cancellous bone exhibits a higher bone turnover compared to cortical bone. Fatigue loading induces formation of microcracks in areas of cortical bone which are subsequently resorbed (Verborgt et al., 2000). Furthermore, it was shown that both mechanical disuse and fatigue loading increase osteocyte apoptosis in specific bone regions (Verborgt et al., 2000; Aguirre et al., 2006). Indeed, tail-suspension stimulates osteocyte apoptosis, which is followed by bone resorption targeted to areas containing the apoptotic osteocytes in mice (Aguirre et al., 2006).

Bone remodelling has been also shown to play a key role in calcium and phosphorus homeostasis (Peterson and Riggs, 2010). The osteoclastic action that returns bone mineral into the bloodstream is enhanced by the secretion of parathyroid hormone (PTH), which controls calcium homeostasis at several levels and is increased if calcium deficiency is detected in the serum. In such case, calcium must be retrieved from bone matrix, where it is stored. Martínez-Reina et al. (2008) hypothesised that calcium retrieval is potentially more effective if it takes place at highly mineralised bone sites. This fact could be linked to targeted bone remodelling, as highly mineralised bone also accumulates a larger amount of damage (O'Brien et al., 2000; Qiu et al., 2005). Thus, the target of bone remodelling would not only be repairing damage, but also returning calcium to the bloodstream as efficiently as possible.

The mechanobiological link between microdamage and remodelling was established via discovery of increased remodelling around apoptotic osteocytes in rat ulnar fatigue-loading experiments (Verborgt et al., 2000). In the latter case, inhibition of osteocyte apoptosis prevents the intra-cortical resorption that occurs in response to microcracks (Cardoso et al., 2009), suggesting that osteocyte apoptosis controls osteoclast recruitment to the damaged area. Consistent with this idea, Tatsumi et al. (2007) have demonstrated that stimulation of osteocyte apoptosis, in and of itself, is sufficient to stimulate bone resorption that is associated with an increase in RANKL production in bone, but the cellular source of the RANKL was not determined.

There are limited data on bone quality for patients treated with denosumab compared to other anti-resorptive drugs. The pharmacodynamics of denosumab is different to that of other drugs, but it is their anti-resorptive action on bone remodelling that results in similar effects on bone quality. A recent study on the use of bisphosphonate (BP) treatment showed that the anti-resorptive therapy did not result in a detectable mechanical benefit in the trabecular bone specimens (from hip fracture patients) examined (Jin et al., 2017). Instead, BP use was associated with substantially reduced bone strength. This low strength may be due to the greater accumulation of microcracks and a lack of any discernible improvement in bone volume or microarchitecture. That study suggested that the clinical impact of BP-induced microcrack accumulation may be significant. Major current limitations on detecting significant effects of anti-resorptive treatments on microcrack density in bone is due to the fact that bone biopsies are only taken at non-load bearing bone sites, e.g., iliac crest, hence, masking the effect of mechanical loading and anti-resorptive therapy. However, it is very plausible from a material science point of view to hypothesise that a higher mineralised bone matrix (as observed with anti-resorptive treatment) will accumulate more microcracks during dynamic loading.

Based on the above described mechanisms observed for action of denosumab treatment of PMO, we have developed a comprehensive model of bone remodelling incorporating the effect of bone mineralisation, microdamage, and mechanobiological feedback. The current model of bone remodelling is an extension of our previous mechanistic PK-PD model of the effects of denosumab treatment on PMO (Martínez-Reina and Pivonka, 2019; Martínez-Reina et al., 2021) with respect to accounting for the accumulation of microdamage in the bone matrix. This model incorporates the relation between bone turnover and BMD, together with the bone mineralisation process and the formation of microdamage in the bone matrix. The evolution law for damage accumulation is formulated within the framework of continuum damage mechanics (Lemaitre and Chaboche, 1990). The bone remodelling model accounts for bone cell interactions via the RANK-RANKL-OPG pathway, the action of TGF–β and mechanobiological feedback (Martínez-Reina and Pivonka, 2019; Martínez-Reina et al., 2021; Pivonka et al., 2008, 2010; Scheiner et al., 2013). Mechanical overuse is simulated via increase of osteoblast precursors proliferation (Pivonka et al., 2012; Scheiner et al., 2013, 2014), while mechanical disuse is simulated via RANKL production by osteoblast precursor cells. Furthermore, bone matrix damage was linked to increased RANKL production by (apoptotic) osteocytes. The mineralisation model takes into account the balance of mineral within bone tissue and is based on the work of Martínez-Reina et al. (2008). As in previous studies, the PK model of denosumab is a one compartment model including a drug saturation term for high doses (see Marathe et al., 2011).

Utilising this model, we investigate a variety of treatment scenarios with emphasis on combined effects of mechanical loading (including overuse and disuse) together with denosumab treatment in PMO.

## 2. Mechanistic PK-PD Model of Bone Adaptation Including Damage

### 2.1. Model of Bone Cell Interactions in Bone Adaptation

A brief description of the mathematical model describing bone cell interactions is provided. As in previous models, the RANK-RANKL-OPG pathway, together with the action of several regulatory factors on bone cells, including TGF–β, PTH, and mechanobiological feedback is given (for details on original models see Pivonka et al., 2008, 2010; Scheiner et al., 2013; Pivonka et al., 2012; Martínez-Reina and Pivonka, 2019). The new model has been designed following the structure of the original model, adding the population of osteocytes, as done in Martin et al. (2019), slightly modifying the mechanoregulation feedback and adding new features relevant to the formulation of damage, the last two modifications being dealt with in subsections 2.3 and 2.4, respectively.

**Figure 1:**
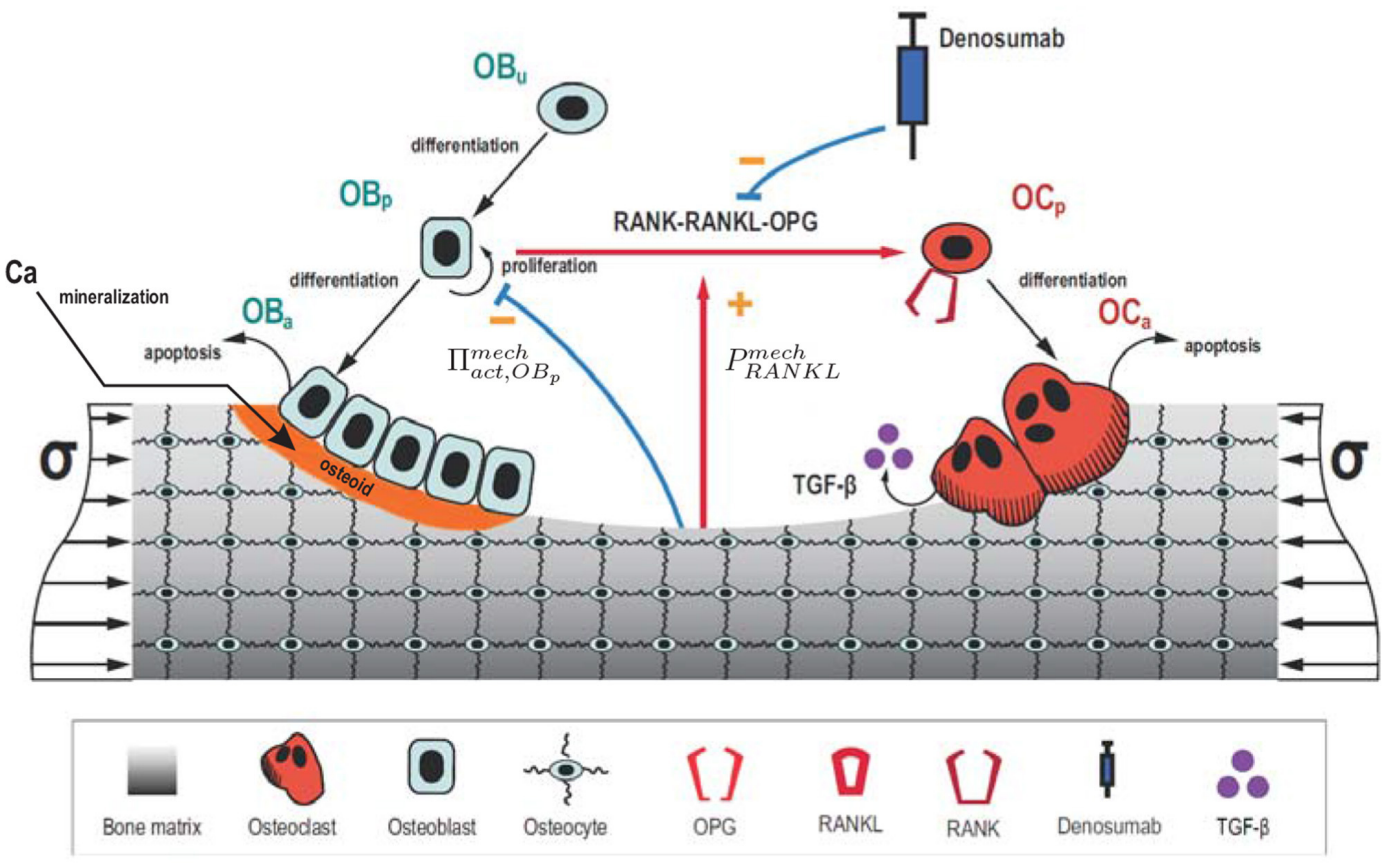
Schematic representation of the mechanistic PK-PD model: bone cell differentiation stages along with biochemical and biomechanical interactions are presented. Subcutaneous injection of denosumab leads to distribution of the drug into the central compartment where it interacts with the RANK-RANKL-OPG pathway (red arrow between *Ob*_*p*_ and *Oc*_*p*_). The latter interactions are accounted for via competitive binding reactions. The mineralisation of osteoid is shown in orange.

Following the approach taken by Pivonka et al., the bone adaptation process can be described as cell balance equations. The bone cell types (i.e., state variables) considered in the current model are: (i) osteoblast precursor cells (Ob_p_), (ii) active osteoblasts (Ob_a_), (iii) active osteoclasts (Oc_a_), and (iv) osteocytes (Ot). The cell pools of uncommitted osteoblasts (Ob_u_) and osteoclast precursors (Oc_p_) are assumed constant:

(1) $$\begin{array}{l} \begin{array}{c} \frac{dOb_{p}}{dt}=D_{Ob_{u}}\cdot \Pi_{act}^{TGF-\beta }\cdot Ob_{u}+P_{Ob_{p}}\cdot \Pi_{act}^{\psi_{bm}}\cdot Ob_{p}\\ \;-D_{Ob_{p}}\cdot \Pi_{rep}^{TGF-\beta }\cdot Ob_{p}; \end{array} \end{array}$$

(2) $$\begin{array}{l} \frac{dOb_{a}}{dt}=D_{Ob_{p}}\cdot \Pi_{rep}^{TGF-\beta }\cdot Ob_{p}-A_{Ob_{a}}\cdot Ob_{a}; \end{array}$$

(3) $$\begin{array}{l} \frac{dOc_{a}}{dt}=D_{Oc_{p}}\cdot \Pi_{act}^{RANKL}\cdot Oc_{p}-A_{Oc_{a}}\cdot \Pi_{act}^{TGF-\beta }\cdot Oc_{a}; \end{array}$$

(4) $$\begin{array}{l} \frac{dOt}{dt}=\eta \frac{df_{bm}}{dt} \end{array}$$

where *D*_*Ob*_*u*__, *D*_*Ob*_*p*__, and *D*_*Oc*_*p*__ are the differentiation rates of Ob_u_, Ob_p_, and Oc_p_ respectively. The second term in the right-hand side of Equation (1) corresponds to proliferation of osteoblast precursors and *P*_*Ob*_*p*__ gives the maximum proliferation rate. *A*_*Ob*_*a*__ and *A*_*Oc*_*a*__ are the apoptosis rates of Ob_a_ and Oc_a_ respectively. The variables $\Pi_{act}^{TGF-\beta }$, and $\Pi_{rep}^{TGF-\beta }$ represent activator and repressor functions related to the binding of TGF–β to its receptor. Similarly, $\Pi_{act}^{RANKL}$ is the activator function related to the RANK-RANKL binding. $\Pi_{act}^{\psi_{bm}}$ is a function of the mechanical stimulus that regulates the anabolic part of the mechanobiological feedback in the proliferation term and will be addressed in section 2.3. Finally, η is the concentration of osteocytes in bone matrix, which is assumed constant as in Martin et al. (2019), thus leading to proportional variations of osteocytes population and fraction of bone matrix volume per total volume, *f*_*bm*_ (Equation 4). The variation of *f*_*bm*_ over time is precisely one of the main outcomes to be derived from the set of cell population equations and is defined through the balance between resorbed and formed tissue:

(5) $$\begin{array}{l} \frac{df_{bm}}{dt}=-k_{res}\cdot Oc_{a}+k_{form}\cdot Ob_{a}; \end{array}$$

where *k*_*res*_ and *k*_*form*_ are, respectively, the bone matrix volume resorption rate and osteoid volume formation rate. This distinction is important with regard to the mineralisation algorithm, since the bone matrix resorbed by active osteoclasts is mineralised, while the osteoid deposited by active osteoblasts contains no mineral. Cell balance equations (Equations 1–4) are composed of a production term and a degradation one, which describes differentiation of one cell type into another (or terminal cell fate, i.e., apoptosis). A schematic figure of the mechanistic PK-PD model is presented in Figure 1. Model parameters of the cell population model are given in **Table A1**.

### 2.2. Denosumab Action on RANK-RANKL-OPG: Competitive Binding

The action of denosumab on bone adaptation is included via competitive binding reactions within the RANK-RANK-OPG pathway (Marathe et al., 2008, 2011; Scheiner et al., 2014). In these models, action of denosumab is taken into account via the RANKL activator function $\Pi_{act}^{RANKL}$ (Equation 3, first term on the right). Denosumab competes with RANK (and OPG) for binding to RANKL. Thus, higher concentrations of denosumab give rise to lower concentrations of RANKL-RANK complexes and, hence, lower values of $\Pi_{act}^{RANKL}$. Adapting the approach of Scheiner et al. (2014),

(6) $$\begin{array}{l} RANKL=RANKL_{eff}\frac{\beta_{RANKL}+P_{RANKL}}{\beta_{RANKL}+{\tilde{D}}_{RANKL}RANKL_{eff}}\cdot \\ \cdot [1+\frac{OPG}{K_{d,\left[RANKL-OPG\right]}}+\frac{RANK}{K_{d,\left[RANKL-RANK\right]}}\\ \;+\frac{\zeta C_{p,den}}{K_{d,\left[RANKL-den\right]}}{]}^{-1}; \end{array}$$

where *K*_*d*, [*RANKL*−*OPG*]_, *K*_*d*, [*RANKL*−*RANK*]_, and *K*_*d*, [*RANKL*−*den*]_ are the equilibrium dissociation binding constants for binding of OPG, RANK and denosumab to RANKL. *OPG*, *RANK*, and *RANKL* are the concentrations of respective regulatory factors in the bone tissue compartment, while *C*_*p,den*_ is the concentration of denosumab in the central compartment (see Equation 36 in Appendix) and ζ is the accessibility factor of denosumab from the central compartment to the bone tissue compartment^1^. In the original model (Lemaire et al., 2004) all concentrations were formulated with respect to a pseudo central compartment and, consequently, no distinction between site-specific bone tissue compartments was needed. However, formulation of mechanobiological PK-PD models requires specification of a particular bone site which is exposed to physiological mechanical loading. ${\tilde{D}}_{RANKL}$ is the RANKL degradation rate, *P*_*RANKL*_ provides the RANKL production rate induced by PMO and mechanical underloading. β_*RANKL*_ is the production rate of endogenous RANKL on the surface of osteoblasts precursors and osteocytes. We have assumed that RANKL is expressed by those cells, following experimental evidence (Nakashima et al., 2011; Xiong and O'Brien, 2012) and a previous model (Martin et al., 2019). So, *RANKL*_*eff*_ is the total effective carrying capacity of those cells that controls the maximum expression of RANKL:

(7) $$\begin{array}{l} RANKL_{eff}=R_{RANKL}\cdot Ob_{p}\cdot \Pi_{act}^{PTH}+R_{RANKL}\cdot Ot\cdot \Pi_{act}^{dam} \end{array}$$

where *R*_*RANKL*_ is the carrying capacity of the individual cells of both types, that we have assumed equal. We have also assumed that the expression of RANKL on the surface of osteoblast precursors is upregulated by PTH, following previous models (Pivonka et al., 2008, 2010), and we have introduced in the present model an upregulation factor of RANKL expression by osteocytes due to damage, through a sigmoidal function:

(8) $$\begin{array}{l} \Pi_{act}^{dam}=\frac{d^{\xi }}{d^{\xi }+\delta_{50}^{\xi }} \end{array}$$

where *d* denotes the damage variable, described more in detail in section 2.4. The shape factor, ξ = 3, and the value of damage leading to a 50% of the maximum response, δ_50_ = 0.1, were chosen in such a way that both terms in Equation 7 are typically of the same order of magnitude^2^. Verborgt et al. (2000) showed that osteocyte apoptosis occurs after fatigue induced bone matrix damage. Moreover, they found that osteocyte apoptosis was highly localised to sites of microdamage that are subsequently remodelled. Osteocytes in the vicinity of a microcrack would express both Bax (a proapoptotic gene product) and Bcl-2 (an antiapoptotic gene product), with the peak of Bax expression observed immediately at the microcrack locus and the peak of Bcl-2 expression at some distance (1–2 mm) from microcracks (Verborgt et al., 2002). Seemingly, distant osteocytes would protect themselves from matrix injury induced cell death, thereby exercising an additional level of control in the regulation of osteocyte apoptosis and bone remodelling. This expression of apoptotic signals would be related to the expression of *come and eat me* signals (Jin and El-Deiry, 2005) to attract macrophages to the site of apoptotic osteocytes. Kurata et al. (2006) showed later that focal wounding of osteocyte-like cells (MLO-Y4) *in vitro* triggered release of RANKL and macrophage-colony stimulating factor (M-CSF), although whether these key signaling molecules come from dying cells or the non-apoptotic surviving cells was not examined. Here we have assumed by using Equation (7) that apoptotic osteocytes near microcracks would express RANKL so causing their surrounding bone matrix to be resorbed.

In Equation (6), the RANKL production rate *P*_*RANKL*_ is given by two terms that define the contribution of mechanical underloading, $P_{RANKL}^{mech}$, and a disease-related increase in RANKL production over time, $P_{RANKL}^{PMO}$:

(9) $$\begin{array}{l} P_{RANKL}=P_{RANKL}^{mech}+P_{RANKL}^{PMO} \end{array}$$

The first term is explained in section 2.3, while the term $P_{RANKL}^{PMO}$ is a consequence of the onset of menopause though increasing gradually over time, through the following sigmoidal function:

(10) $$\begin{array}{l} P_{RANKL}^{PMO}\left(t\right)=P_{RANKL}^{PMO,max}\frac{{\left(t-t_{onset}\right)}^{2}}{{\left(t-t_{onset}\right)}^{2}+\delta_{PMO}^{2}}\text{ for}t>t_{onset} \end{array}$$

where $P_{RANKL}^{PMO,max}$ is the maximum (long-term) RANKL production rate due to PMO, *t*_*onset*_ is the time of onset of the disease and δ_*PMO*_ is a time constant that establishes when the 50% of $P_{RANKL}^{PMO,max}$ is reached.

Finally, the activator function of RANKL in Equation (3) can be expressed as:

(11) $$\begin{array}{l} \Pi_{act}^{RANKL}=\frac{RANKL\cdot RANK}{K_{d,\left[RANKL-RANK\right]}+RANKL\cdot RANK}; \end{array}$$

The concentrations of denosumab, *RANK*, *OPG*, *PTH*, and *TGF*−β along with the model parameters of the binding reactions are provided in the Appendix.

### 2.3. Mechanoregulation

**Figure 2:**
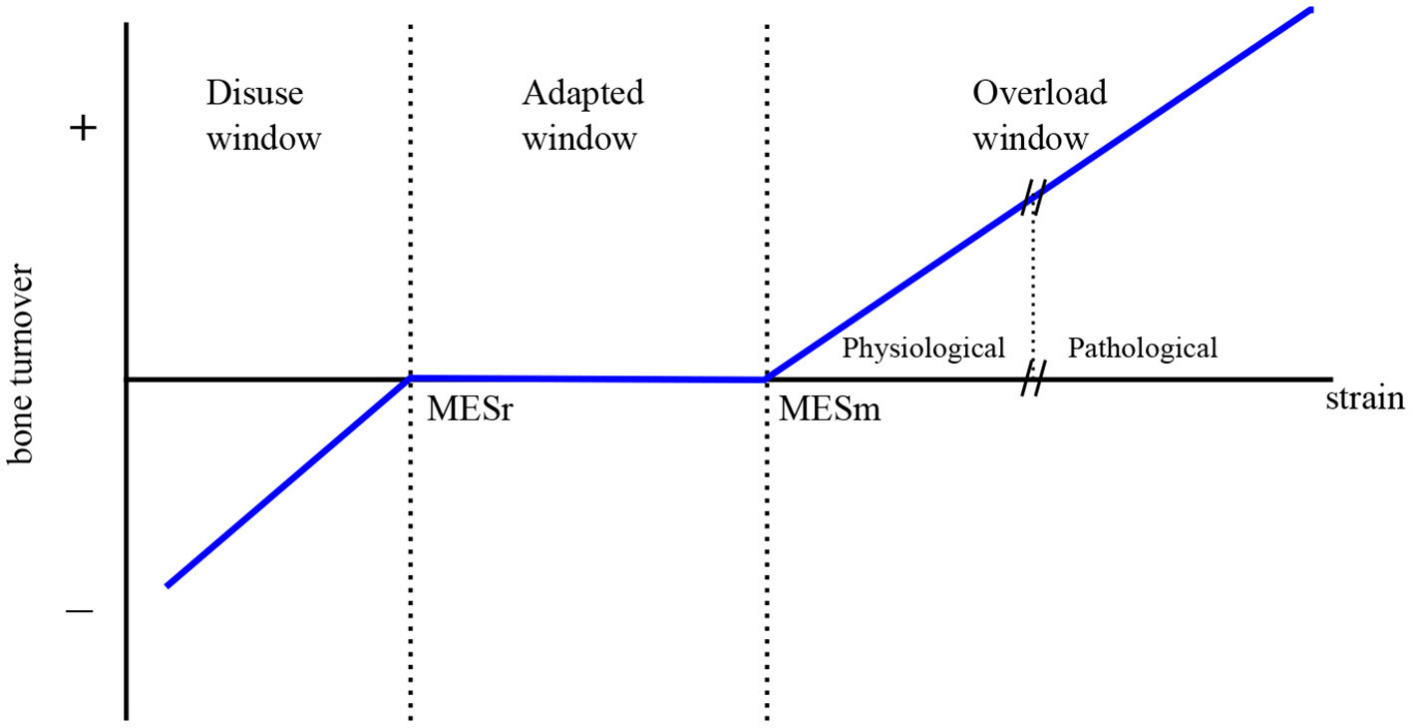
The Mechanostat Theory (adapted from Frost, 2003).

**Figure 3:**
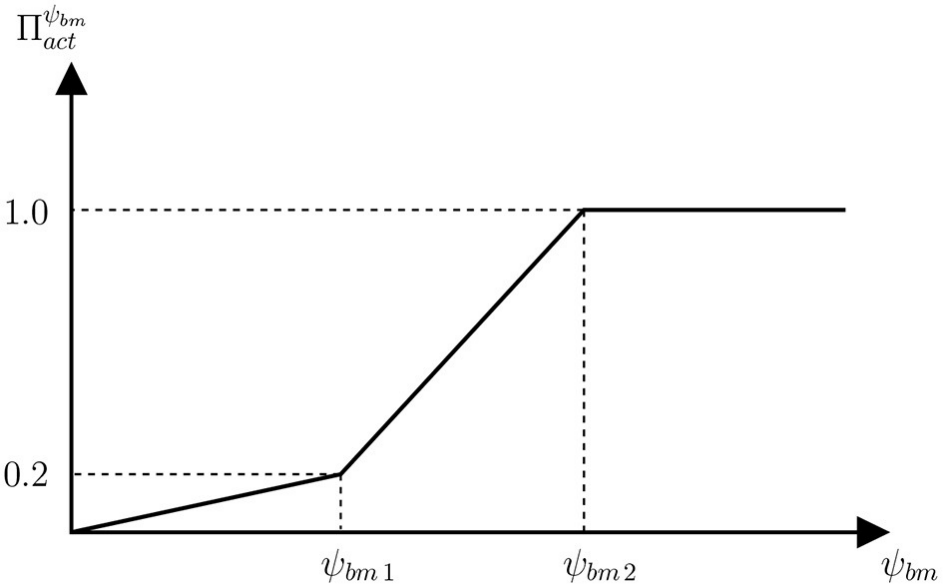
Function of proliferation of osteoblasts precursors that establishes the relation between the anabolic factor $\Pi_{act}^{\psi_{bm}}$ and the SED.

The model includes the mechanical feedback regulation of bone through the Mechanostat Theory proposed by Frost (2003) (see Figure 2). This theory postulates the existence of 4 zones or “windows” in Frost's terminology: (1) disuse window, where net bone loss is observed for a low level of “Minimally Effective Strains” (MES) or other stimuli; (2) adapted window, where no net effect of BMUs on bone mass is seen for intermediate values of MES; (3) mild overload window, where net bone formation occurs for high MES, and (4) pathologic overload window, leading to fracture, for very high values of MES. This last window is not directly considered in the mechanical regulation through the definition of $\Pi_{act}^{\psi_{bm}}$ (see Figure 3), but indirectly through the accumulation of microstructural damage.

Mechanical disuse is assumed to enhance the production of RANKL on osteoblasts precursors, through the term $P_{RANKL}^{mech}$, which is modulated by the strain energy density (SED) of bone matrix, designated as ψ_*bm*_:

(12) $$\begin{array}{l} P_{RANKL}^{mech}=\{\begin{array}{ll} P_{RANKL}^{mech,max}\left(1-\frac{\psi_{bm}}{\psi_{r}}\right) & \text{for }\psi_{bm}<\psi_{r}\\ 0 & \text{for }\psi_{bm}\geq \psi_{r} \end{array} \end{array}$$

where ψ_*r*_ is the SED below which underuse increases RANKL production and $P_{RANKL}^{mech,max}$ is the maximum RANKL production rate due to underuse. RANKL production is upregulated by PTH and downregulated by nitric oxide (NO), which is produced by osteocytes and, in turn, upregulated by mechanical stimulus. However, this process is only indirectly considered through Equation (12), which assumes a maximum RANKL production rate for total disuse.

Overload is assumed to promote bone formation by proliferation of osteoblasts precursors through the activator function $\Pi_{act}^{\psi_{bm}}$, which is given by the piecewise linear function of SED defined in Figure 3. The less steep piece of the function would correspond to the disuse and adapted windows of the Mechanostat Theory, where bone formation is not particularly promoted. In the case of the disuse window, this would be added to the increased RANKL production ($P_{RANKL}^{mech}$). Obviously, the steepest piece of function $\Pi_{act}^{\psi_{bm}}$ would correspond to the overload window.

The SED, ψ_*bm*_, was used as a measure of the mechanical stimulus sensed by bone cells to drive bone adaptation, as traditionally done in the literature (Beaupré et al., 1990; Huiskes et al., 1987). ψ_*bm*_ was used here as an alternative to the strains *MES*, used in the Mechanostat Theory (Frost, 2003). In a uniaxial stress state they are related by:

(13) $$\begin{array}{l} \psi_{bm}=\frac{1}{2}E\cdot MES^{2} \end{array}$$

being *E* the Young's modulus. The parameter ψ_*r*_ in Equation (12) as well as ψ_*bm*1_ and ψ_*bm*2_, used in Figure 3 to define function $\Pi_{act}^{\psi_{bm}}$, were defined using Equation (13), respectively with *MES*_*r*_ = 1,000 με, *MES*_1_ = 800 με, and *MES*_2_ = 1,600 με. These values and the values of $\Pi_{act}^{\psi_{bm}}$ corresponding to ψ_*bm*1_ and ψ_*bm*2_ were adjusted to reproduce the Mechanostat Theory along with the Principle of Cellular Accomodation (see Appendix).

### 2.4. Damage of Bone Matrix

In this section we address how to estimate microstructural damage, that we assumed to drive bone remodelling through Equation (7) and to affect mechanical properties as will be discussed shortly. It has long been hypothesised that one of the major functions of bone remodelling is to remove microcracks from the bone matrix and so to avoid accumulation of the latter, which could result in macroscopic failure. One way to describe the accumulation of microcracks in a particular volume of material is via use of Continuum Damage Mechanics (Lemaitre and Chaboche, 1990). The latter theory introduces a damage variable, *d*, which is linked to the density of microcracks in a volume of material and to the loss of stiffness through Equation (14). This is variable is such that *d* ∈ [0, 1], with *d* = 0 corresponding to an undamaged state and *d* = 1 to a local fracture or failure situation:

(14) $$\begin{array}{l} \text{C}=\left(1-d\right){\text{C}}_{0} \end{array}$$

where **C** and **C**_0_ are, respectively, the stiffness tensors of damaged and undamaged bone (Lemaitre and Chaboche, 1990)^3^.

Microdamage accumulates in the bone matrix due to fatigue loading and is repaired by bone remodelling, as osteoclasts resorb the damaged tissue while the osteoid deposited by osteoblasts is initially intact. The evolution law for damage can be expressed as:

(15) $$\begin{array}{l} \dot{d}={\dot{d}}_{A}-{\dot{d}}_{R} \end{array}$$

where $\dot{d}$_*A*_ is the rate of damage accumulation by fatigue loading and $\dot{d}$_*R*_ is the rate of damage removal by bone remodelling. The latter is assessed by assuming that damage is uniformly distributed throughout the representative volume element (RVE). Thus, the amount of damage repaired by remodelling is proportional to the damage present in that volume and to the volume of tissue being resorbed (see Equation 5), through the fraction that this volume represents within the bone matrix volume:

(16) $$\begin{array}{l} {\dot{d}}_{R}=d\frac{k_{res}\cdot Oc_{a}}{f_{bm}} \end{array}$$

Damage accumulation is evaluated following the procedure described in Martínez-Reina et al. (2008) and Martínez-Reina et al. (2009), which, in turn, are based on the works by Pattin et al. (1996) and García-Aznar et al. (2005). Experimental fatigue tests provide the evolution of *d* with the strain or the stress level and the number of cycles (Pattin et al., 1996), as well as fatigue life, *N*_*f*_, which is typically given by expressions such as:

(17) $$\begin{array}{l} \begin{array}{cc} N_{f}=\frac{K_{i}}{\varepsilon^{\delta_{i}}} & \;i=c\left(\text{compresion}\right),t\left(\text{tension}\right) \end{array} \end{array}$$

where *K*_*i*_ and δ_*i*_ stand for constants that are different in tension and compression and ε is the uniaxial strain expressed in με = μ*m*/*m*. García-Aznar et al. (2005) correlated Equation (17) with the experimental results obtained by Pattin et al. (1996) to get: $K_{c}=9.333\cdot 10^{40}$ and δ_*c*_ = 10.3 in compression, and $K_{t}=1.445\cdot 10^{53}$ and δ_*t*_ = 14.1 in tension. The loss of stiffness *E*/*E*_0_ was also experimentally measured by those authors as a function of the applied constant strain and the number of cycles. Again, García-Aznar et al. (2005), fitted the experimental curves obtained by Pattin and co-workers with the following expressions:

(18a) $$\begin{array}{l} d_{c}=-\frac{1}{\gamma_{c}}\left[\ln\;\left(1-C_{c}\varepsilon^{\delta_{c}}N\right)\right] \end{array}$$

(18b) $$\begin{array}{l} d_{t}=1-{\left[\frac{1}{C_{t2}}\ln\;\left(e^{C_{t2}}-C_{t1}\varepsilon^{\delta_{t}}N\right)\right]}^{\frac{1}{\gamma_{t}}} \end{array}$$

where *N* is the number of cycles and *C*_*t*2_ = −20 was fitted from the experimental curves along with:

(19) $$\begin{array}{l} \gamma_{c}=-5.238\left(\varepsilon -6100\right)10^{-3}+7;\\ \;C_{c}=\frac{1-e^{-\gamma_{c}}}{K_{c}}\text{in compression}\\ \gamma_{t}=-0.018\left(\varepsilon -4100\right)+12;\;C_{t1}=\frac{e^{C_{t2}}-1}{K_{t}}\text{in tension} \end{array}$$

In the damage model proposed by Martínez-Reina et al. (2009), cracks were assumed to grow normal to the maximum strain direction and only under tensile strains. This allows to apply the model to a general strain state, by replacing ε with the maximum principal strain, ε_*max*_. The tests performed by Pattin et al. (1996) and fitted with Equation (18) were conducted under constant strain. However, they can be applied to a general loading history using the procedure described in Martínez-Reina et al. (2009) and explained next.

Let us assume that, at a given moment, damage is equal to *d* and a maximum principal strain ε_*max*_ is applied *N* cycles in the next step. Let us calculate the increment of damage accumulated by fatigue, Δ*d*_*A*_, with those cycles. Likely, the current damage was not produced by a constant strain ε_*max*_, but we can assume that it was so without loss of generality. Then, we can use Equation (18b) to work out the number of cycles Ñ that would have been needed to reach the current damage *d* with the current strain ε_*max*_.

(20) $$\begin{array}{l} d=1-{\left[\frac{1}{C_{t2}}\ln\;\left(e^{C_{t2}}-C_{t1}\varepsilon_{max}^{\delta_{t}}Ñ\right)\right]}^{\frac{1}{\gamma_{t}}}\;\Rightarrow \;Ñ \end{array}$$

The increment of damage Δ*d*_*A*_ would have been reached with the additional *N* cycles applied at the present step and can be assessed from:

(21) $$\begin{array}{l} d+\Delta d_{A}=1-{\left[\frac{1}{C_{t2}}\ln\;(e^{C_{t2}}-C_{t1}\varepsilon_{max}^{\delta_{t}}\left(Ñ+N\right))\right]}^{\frac{1}{\gamma_{t}}} \end{array}$$

This procedure allows working out the increment of damage, Δ*d*_*A*_, but requires that Equation (15) be rewritten in incremental form and integrated using an explicit integration scheme, as done in Martínez-Reina and Pivonka (2019) and Martínez-Reina et al. (2021).

### 2.5. Degradation of Fatigue Properties With the Mineral Content

The mineral phase contributes to increase the stiffness of bone, but also makes it more brittle (Currey, 2004). As far as we know, no experimental study has provided a correlation between mineral content and bone fatigue properties, though some studies have confirmed that interstitial bone, with the highest mineral content, is where microcracks can be more easily found (Boyce et al., 1998; O'Brien et al., 2000; Qiu et al., 2005). For this reason, we have followed the damage model previously proposed by Martínez-Reina et al. (2009) in which fatigue properties are degraded as the mineral content rises. According to this idea the following assumptions are made in the model (see Martínez-Reina et al., 2009 for more details):

1. The shape of *d* − *N* curves, expressed by the Equations (18), is maintained regardless of the mineral content.
2. Only the fatigue life is affected by the mineral content, by redefining *K*_*t*_ in Equation (17), while keeping constant the exponent δ_*t*_. This modifies the *d* − *N* law, as *C*_*t*1_ depends on *K*_*t*_. In this way, increasing *K*_*t*_ results in a longer fatigue life and a slower damage accumulation rate.
3. A life of 10^7^ cycles was assigned to the fatigue limit. This fatigue limit is usually assumed to occur for a given fraction of the ultimate tensile strain, ε_*u*_/β, where the parameter β may depend on the type of material (Juvinall, 1967). So, *K*_*t*_ was obtained from Equation (17) as:
  (22) $$\begin{array}{l} K_{t}\left(\left[Ca\right]\right)=10^{7}{\left(\frac{\varepsilon_{u}\left(\left[Ca\right]\right)}{\beta }\right)}^{\delta_{t}} \end{array}$$
  Here, the most typical value β = 2 was chosen following Martínez-Reina et al. (2009).
4. As Currey showed (Currey, 2004), ε_*u*_ depends on the calcium concentration of bone matrix, [*Ca*]. The following regression ε_*u*_ = ε_*u*_([*Ca*]) was fitted in Martínez-Reina et al. (2009) from the experimental results presented by Currey (2004):
  (23) $$\begin{array}{l} log\;\varepsilon_{u}=31.452-11.341\;log\;\left[Ca\right] \end{array}$$
  where ε_*u*_ is expressed in με and the concentration [*Ca*] is expressed in mg of calcium per g of bone matrix and is related to the ash fraction, α, the ratio between the ash mass and the dry mass (see Appendix for details). More precisely, the relation [*Ca*] = 398.8 · α was assumed, based on the molecular weigths of hydroxyapatite and type I collagen. Equations (20), (22), and (23) allowed to define *C*_*t*1_ as a function of α to be used in (18b) and the related equations.

The value fitted by García-Aznar et al. (2005), $K_{t}=1.445\cdot 10^{53}$, corresponds to a normal value of ash fraction, α = 0.72. The importance of the degradation of fatigue properties with the mineral content on the risk of local failure will be evaluated in a simulation in which two cases will be compared: (1) a model implementing Equations (22) and (2) a model using $K_{t}=1.445\cdot 10^{53}$ constant.

### 2.6. Bone Apparent Density and Stiffness

Bone apparent density changes as a consequence of the variation of porosity, accounted by Equation (5), and mineralisation. The latter process controls tissue density, ρ_*t*_, given by:

(24) $$\begin{array}{l} \rho_{t}=\frac{m}{V_{bm}}=\rho_{m}v_{m}+\rho_{o}v_{o}+\rho_{w}v_{w} \end{array}$$

where *m* and *V*_*bm*_ are, respectively, the mass and volume occupied by bone matrix, while *v*_*m*_, *v*_*o*_, and *v*_*w*_ stand for the specific volumes of the three phases that compose bone matrix (namely, mineral, organic, and water) and ρ_*i*_ stand for the corresponding densities. While *v*_*o*_ can be assumed constant, *v*_*m*_ and *v*_*w*_ vary throughout the mineralisation process (see Appendix for details). Bone apparent density is then given by porosity (or alternatively bone volume fraction) and bone tissue density:

(25) $$\begin{array}{l} \rho =\frac{m}{V_{bm}}\frac{V_{bm}}{V_{RVE}}=\rho_{t}f_{bm} \end{array}$$

Finally, bone stiffness is needed to assess SED. In this study, we have assumed that bone tissue is an isotropic material with a Poisson's ratio ν = 0.3 and a Young's modulus given in MPa by the following correlations:

(26) $$\begin{array}{l} E\left(\rho ,d\right)=\{\begin{array}{cc} 2014\rho^{2.5}\left(1-d\right) & \text{ if }\rho <1.2g/cm^{3}\\ 1763\rho^{3.2}\left(1-d\right) & \text{ if }\rho \geq 1.2g/cm^{3} \end{array} \end{array}$$

These expressions are based on the correlations experimentally obtained by Jacobs (1994), which are multiplied by the factor (1 − *d*), to consider microstructural damage as usually done in Continuum Damage Mechanics (Lemaitre and Chaboche, 1990) (recall Equation 14).

### 2.7. Estimation of the Risk of Local Failure

The likelihood of osteoporotic patients suffering a fracture depends on many factors, such as: bone apparent density, amount of microstructural damage, mineral content and brittleness of bone matrix, trabecular microarchitecture, magnitude and orientation of the load, among many others. For this reason, evaluating the risk of fracture or the strength of a certain bone is a complex task. Several works have addressed this problem at a structural level, estimating the bone strength and/or failure patterns using a FE modelling approach (Hambli, 2013a,b; Harrison et al., 2013; Fan et al., 2016; Hambli et al., 2016), but it is out of the scope of the present work.

Having recognised this limitation, it would be interesting, however, to have a tool to compare the risk of failure in different scenarios, at least at a local, i.e., material point of view. To this end, we have defined a variable to estimate the risk of failure by taking into account only bone apparent density, damage, mineral content and magnitude of the load from the factors referred to above. In order to define that variable we have used the equations presented in subsection 2.4.

More precisely, we have defined the variable *NDtF*(σ, *t*), which gives the number of days needed to reach local failure (*d* = 1) at a given instant, *t*, if an overload consisting in a uniaxial stress σ is applied from that moment on. We use the variables *f*_*bm*_(*t*), *d*(*t*), *Oc*_*a*_(*t*), and α(*t*) corresponding to that instant and assume that all of them, except damage, remain constant until failure, which is a strong simplification, especially if *NDtF* yields a high value. Next, we define a time variable τ commencing at the instant *t* and describing the evolution that damage would follow until failure if the conditions at time *t* were kept constant, i.e., *d*(*t* + τ). *NDtF* is the number of days that fulfills the following equation *d*(*t* + *NDtF*) = 1.^4^ The algorithm used to assess *NDtF*(σ, *t*) consists of the following steps:

1. Start from *f*_*bm*_(*t*), *d*(*t*), *Oc*_*a*_(*t*), and α(*t*).
2. Apparent density, ρ(*t*), is obtained from *f*_*bm*_(*t*) and α(*t*) (see Appendix).
3. For a given instant *t* + τ, *d*(*t* + τ) is known (commencing with τ = 0 and *d*(*t*)). Thus, evaluate the Young's modulus from Equation (26), so to obtain *E*(ρ(*t*), *d*(*t* + τ)).
4. Assuming a uniaxial stress state, calculate the maximum principal strain:
  (27) $$\begin{array}{l} \varepsilon_{max}=\{\begin{array}{cc} \frac{\sigma }{E\left(\rho \left(t\right),d\left(t+\tau \right)\right)} & \text{if}\sigma \geq 0\\ -\frac{\nu \sigma }{E\left(\rho \left(t\right),d\left(t+\tau \right)\right)} & \text{if}\sigma <0 \end{array} \end{array}$$
5. With *d*(*t* + τ) and ε_*max*_, use Equation (20) to work out Ñ.
6. Equation (15), (16), and (21) are used to update damage when *N* additional cycles (corresponding to Δτ = 1 day) are applied:
  (28) $$\begin{array}{l} \begin{array}{c} d\left(t+\tau +\Delta \tau \right)=1-{\left[\frac{1}{C_{t2}}\ln\;\left(e^{C_{t2}}-C_{t1}\varepsilon_{max}^{\delta_{t}}\left(Ñ+N\right)\right)\right]}^{\frac{1}{\gamma_{t}}}\\ -d\left(t+\tau \right)\frac{k_{res}\cdot Oc_{a}\left(t\right)\cdot \Delta \tau }{f_{bm}\left(t\right)} \end{array} \end{array}$$
7. If *d*(*t* + τ + Δτ) ≥ 0.999, then assign *NDtF*(σ, *t*) = τ + Δτ and exit the algorithm. Otherwise, go to step 3 and commence a new iteration.

It must be emphasised that *NDtF* is related to the risk of local failure (within the RVE) and that the risk of fracture at the organ level is influenced by other factors, such as the microstructure and the distribution of loads, mechanical properties, density, damage and mineral content throughout the bone among others. This makes it necessary to take into account the whole organ, for example with a FE model, in order to assess the true risk of fracture of the specific organ.

### 2.8. Homeostatic Initial Conditions

All the simulations started from an equilibrium point corresponding to a homeostasis situation under physiological (healthy) conditions. The governing equations constitute a set of differential-algebraic equations whose equilibrium points could be assessed by setting the derivatives equal to zero in Equations (1)-(5) and solving the resulting set of algebraic equations. However, the equations that model the mineralisation process are recursive and cannot be solved either in closed form or numerically. Alternatively, the equilibrium point was obtained by running a simulation for a given stress state and letting the system to reach homeostasis in the long-term. For a given stress, initial conditions were arbitrarily set in a first iteration. The values at the end of an iteration were used as the initial conditions in the next one and the process was repeated until convergence (two successive iterations producing differences less than 0.1% in every variable). We have assumed here a uniaxial stress state, which completely defines the homeostatic condition for a given value of the applied stress.

## 3. Results

**Figure 4:**
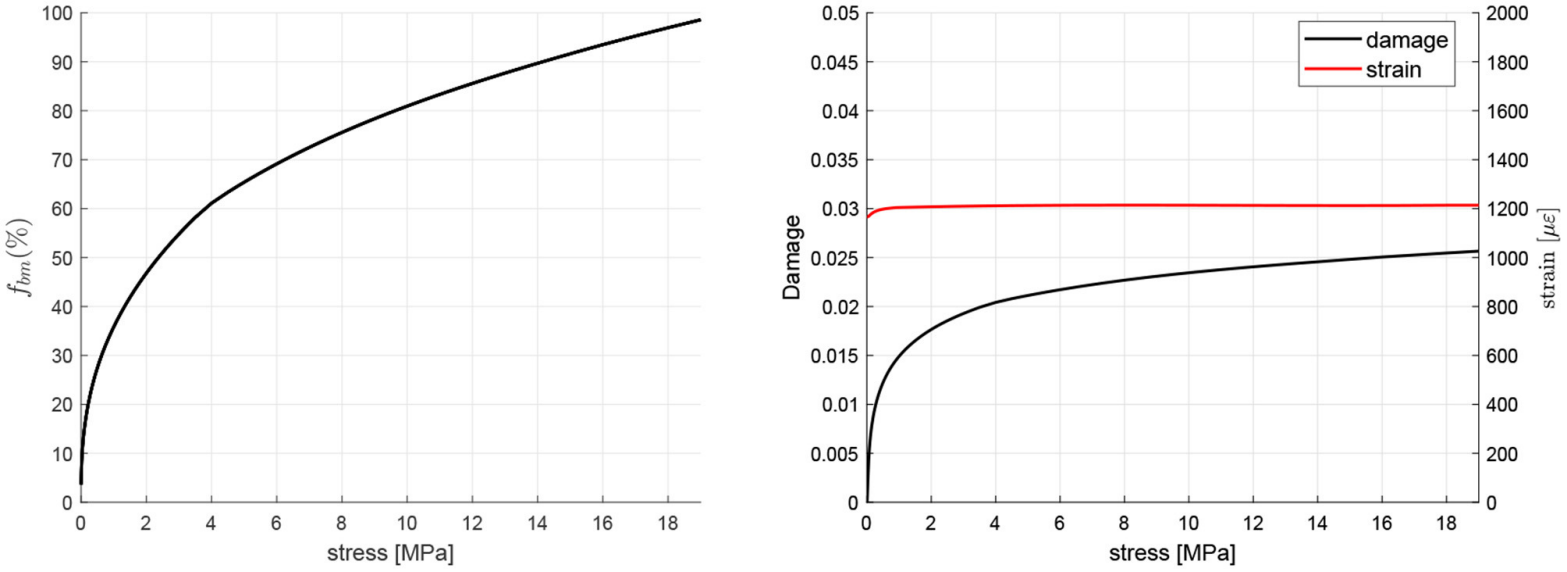
Homeostatic values of *f*_*bm*_, strain and damage *d* for different values of uniaxial tensile stress.

The procedure outlined in subsection 2.8 was used to obtain the equilibrium (homeostatic) points for a range of tensile uniaxial stress. Figure 4 shows the homeostatic values of *f*_*bm*_, strain and damage *d* for each value of the stress. Analogous graphs can be obtained for the rest of variables of the model and for compressive stress.

A tensile stress of 12 MPa was chosen and the homeostatic state for that stress was obtained as explained above. This homeostatic state was used to define the set of initial conditions for all the simulations.

PMO was simulated by a gradual increase in the RANKL production rate (see Equation 10) where the parameter $P_{RANKL}^{PMO,max}$ controls the degree of incidence of the disease, starting from 600 pM day^−1^ to simulate a moderate degree of incidence. The WHO-approved denosumab dosage for the treatment of osteoporosis is 60 mg administered every 6 months (60Q6) via subcutaneous injection, being higher doses restricted for the treatment of other diseases such as multiple myeloma or giant cell tumor. Figure 4 shows the evolution of bone apparent density when the treatment is commenced 3 years after the onset of the disease^5^ and the results are compared with the evolution in a non-treatment group. Patient's body weight (BW) influences the concentration of denosumab present in the central compartment (Marathe et al., 2011) and strongly affects the results. For this reason, a BW = 60 kg has been taken as the reference value.

**Figure 5:**
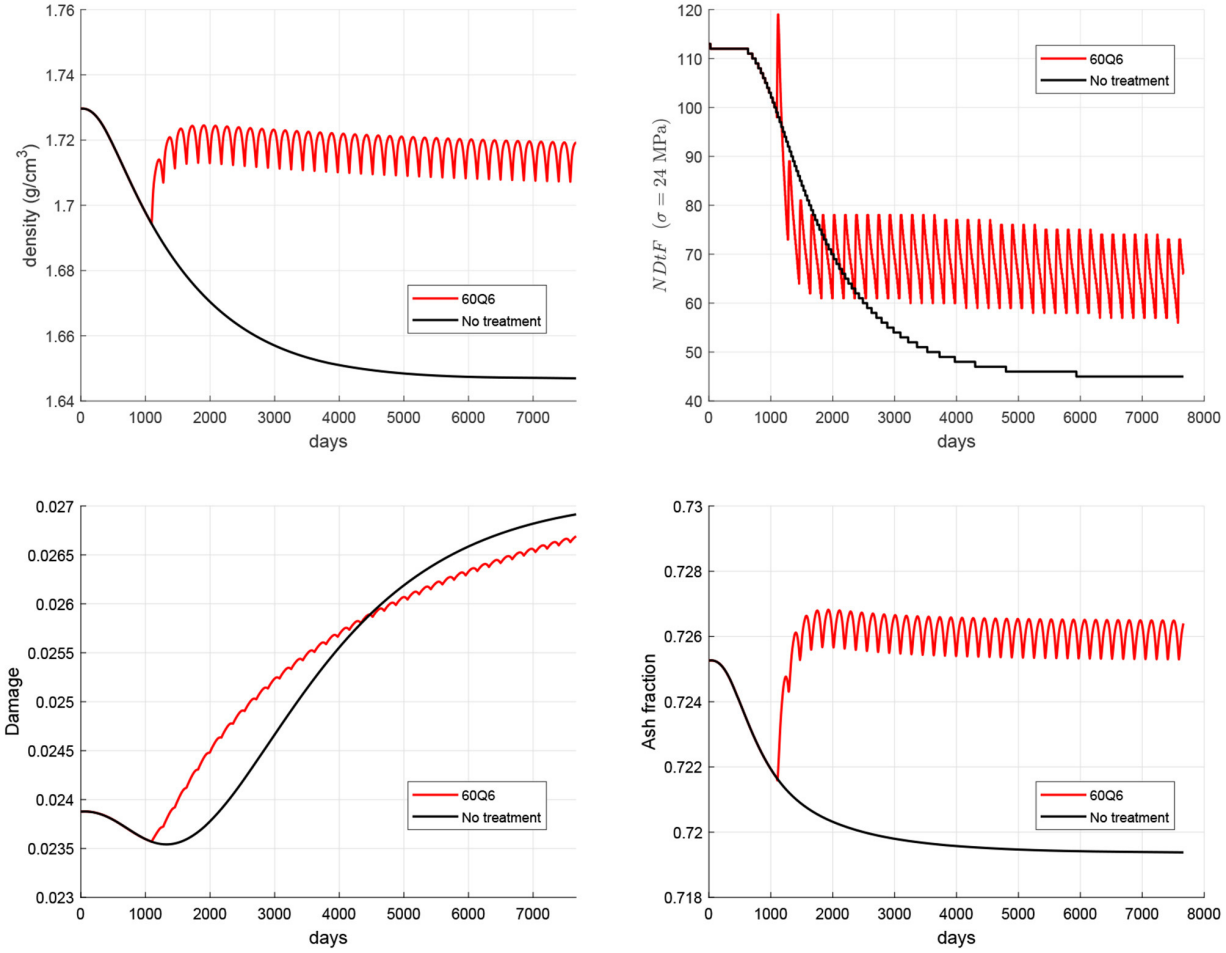
Evolution of density (top left), damage (bottom left), mineral content (bottom right), and NDtF corresponding to an overload σ = 24 MPa (top right) for the WHO-approved treatment 60Q6 and comparison with the non-treatment group.

In order to evaluate the risk of local failure that anti-resorptive treatments may cause in the long-term, the temporal evolution of microstructural damage, ash fraction and *NDtF* are also shown in Figure 5. It can be seen that damage and ash fraction increase simultaneously at the beginning of the treatment. This makes the risk of local failure to rise as well^6^. After a given time, as the mineral content gets stabilised, damage is slowed down and the risk of failure falls below the values obtained for the non-treatment group.

**Figure 6:**
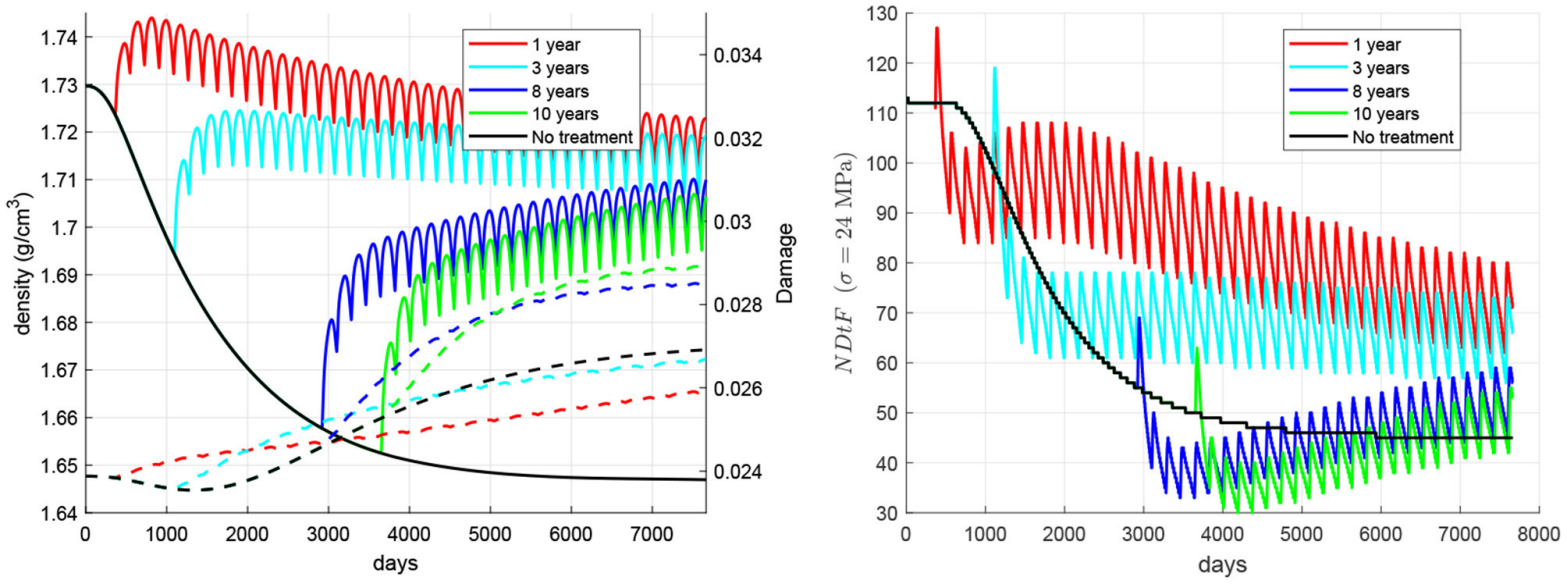
Influence of the time elapsed between the onset of disease and the beginning of the treatment. Evolution of density (left, solid lines), damage (left, dashed lines), and NDtF-σ = 24 MPa (right) for treatment 60Q6.

The time elapsed from the onset of disease to the beginning of the treatment was varied from 1 to 10 years in a set of simulations (see Figure 6) aimed at highlighting the importance of starting the treatment as early as possible. It can be seen how late treatments can notably increase the risk of failure.

**Figure 7:**
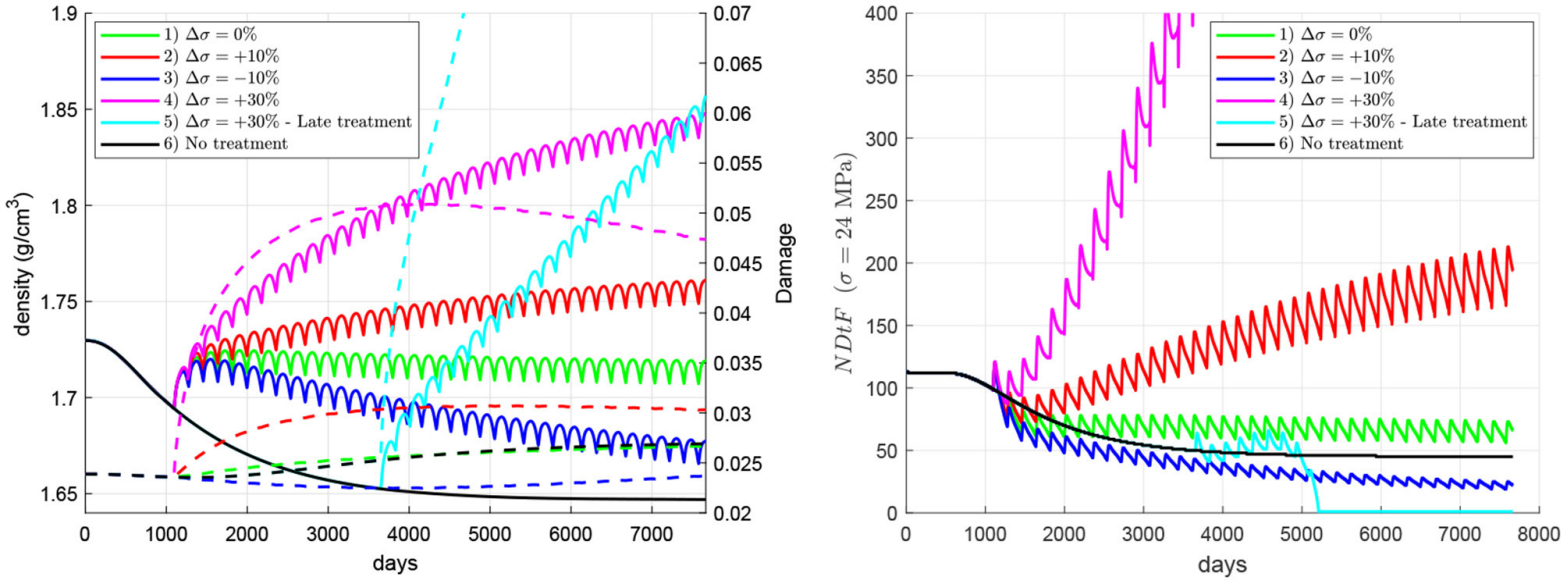
Influence of an increment in the applied stress coinciding with the beginning of the treatment. Evolution of density (left, solid lines), damage (left, dashed lines), and NDtF-σ = 24 MPa (right) for treatment 60Q6. Four cases of changes in the applied stress are compared with the nominal case (no change) and the non-treatment group.

Figure 7 compares the effect of changes in the mechanical loading (i.e., physiological exercise), hereafter denoted as overloading, through the applied stress. Six cases were analysed, all except one (case 6, “no treatment”) including the 60Q6 treatment. In all the cases with treatment, this starts 3 years after the onset of the disease, except for case 5, and the change of load coincides with the beginning of the treatment. The cases are: (1) the nominal case with no change in mechanical load; (2) a stepwise increase of 1.2 MPa (10% of the homeostatic load) in the applied tensile stress; (3) a stepwise decrease of 1.2 MPa; (4) a stepwise increase of 3.6 MPa; (5) a stepwise increase of 3.6 MPa in a late treatment, commencing 10 years after the onset of the disease; (6) no treatment^7^. The results show that bone density rises with the increase of applied stress, though damage can also rise. In this regard, damage can be reduced with a decrease of the applied stress, but this does not imply an improvement of bone quality, since bone density and consequently stiffness may drop significantly, as in case 3, which exhibits a very low *NDtF*. In general, the simulations predicted that an increment of stress coincident with the treatment reduces the risk of local failure, though an excessive increment of stress could be dangerous in a late treatment, in which the initial bone condition can be very deteriorated by the prolonged disease.

**Figure 8:**
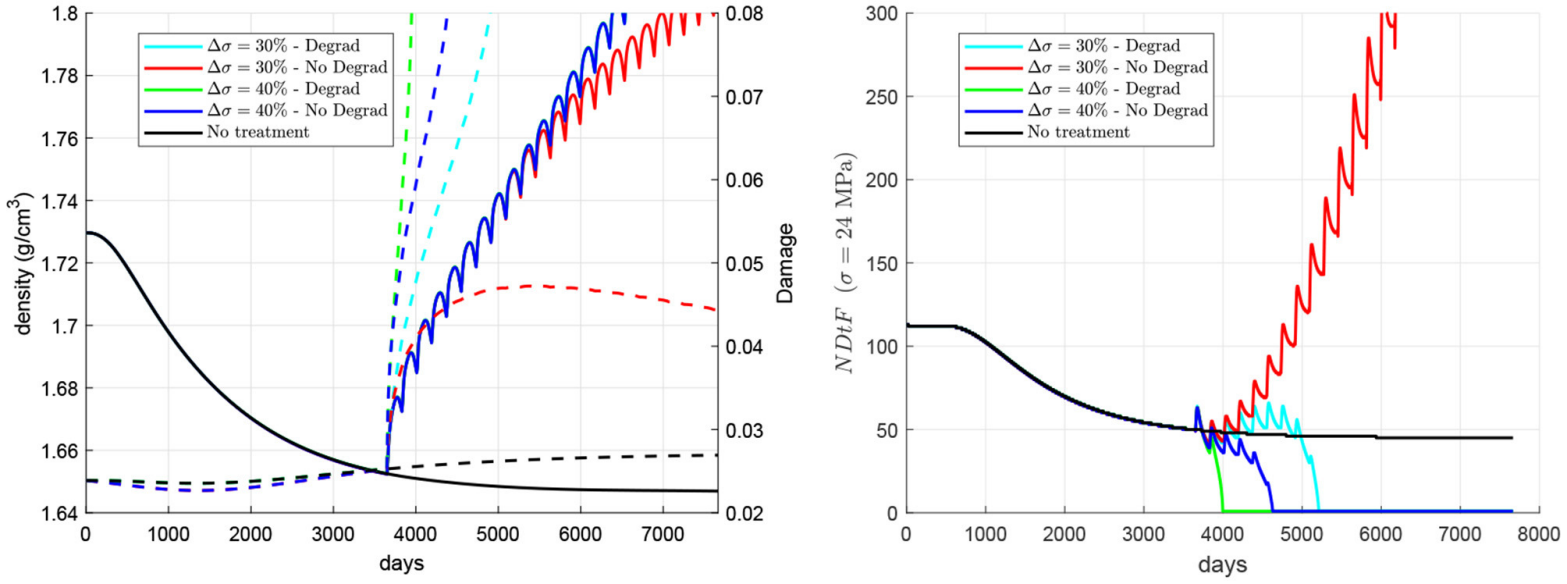
Influence of the degradation of fatigue properties with the mineral content. Evolution of density (left, solid lines), damage (left, dashed lines), and NDtF-σ = 24 MPa (right) for treatment 60Q6. Two scenarios are considered: (a) a stepwise increase of 30% of the nominal stress coinciding with a late treatment and (b) a stepwise increase of 40%; and two models with and without considering the degradation of fatigue properties with the mineral content. Note: the red and blue solid lines in the left figure coincide, respectively, with the cyan and green solid lines.

The importance of accumulation of unrepaired damage and degradation of fatigue properties with the mineral content on the risk of failure is compared next (see Figure 8). To this end, two cases were compared: (1) the model implementing the degradation of fatigue properties through Equation (22), as in the previous simulations, and (2) a model using $K_{t}=1.445\cdot 10^{53}$ constant. In the latter, only the accumulation of unrepaired damage plays a role; while in the former, both factors are important. This will be done for a late treatment 60Q6 beginning 10 years after the onset of the disease and coinciding with a stepwise increase of stress of two different magnitudes: (a) 3.6 MPa (30% of the nominal value) and (b) 4.8 MPa (40%). It can be seen that the embrittlement of bone matrix due to an excessive mineral content plays a key role in the failure experienced with a 30% overload, as the simulation without the degradation of properties does not predict that failure. However, in the case of a 40% overload, the accumulation of unrepaired damage seems to play a more important role, as the simulation without the degradation of properties also predicts that failure.

## 4. Discussion

### 4.1. General Comments

Hernandez et al. (2001b) developed a computational model of bone remodelling to compare the contributions of focal bone balance and mineralisation on BMD by simulating alendronate treatment using a bone balance method (decreased remodelling space, increased focal bone balance, uniform bone mineralisation) and a mineralisation method (decreased remodelling space, neutral focal bone balance, varying bone mineralisation). Their results suggested that the mineralisation method is more descriptive of long-term alendronate treatment indicating that adequate modelling of the mineralisation process is essential to explain observed BMD changes caused by alendronate. While the authors suggest that this model may be used to identify improved dosing regimens and to predict which osteoporosis treatments are more effective, we note that the latter model did not include mechanical loading and microcrack formation explicitly. Hence, the combined effects of physical exercise and treatment with anti-resorptive agents cannot be adequately investigated.

A previous study by Nyman et al. addressed a similar question as we posed in the current paper, i.e., how does mechanical loading and long-term anti-resorptive treatment affect BMD and risk of fracture (Nyman et al., 2004). They utilised a bone remodelling model incorporating micro-damage formation due to mechanical loading together with simulating effects of bisphosphonate treatment. Both disuse and fatigue microdamage were assumed to stimulate the activation frequency of basic multicellular units (BMUs) such that bone remodelling served to remove excess bone mass and microdamage. Bisphosphonate effects were simulated as suppression of BMU activation frequency either without a change in resorption by the BMU or with an independent decrease in resorption while the bone formation process was unaffected (i.e., formation initially exceeded resorption). We note that the initial increase of bone formation with respect to resorption is only temporarily and a very small contributor to increase in BMD in the long term (Martínez-Reina and Pivonka, 2019; Martínez-Reina et al., 2021). Based on the fact that the work of Nyman et al. did not include the mineralisation process they had to include the overfilling of resorption cavities as a mechanism of action of bisphosphonates in order to obtain BMD increases. Their model predicted a plateau in the bone mass gain that typically occurs in clinical studies of bisphosphonate treatment. However, the mechanism of overloading and the mineralisation process were not incorporated into that model.

The above limitations are overcome in the present study in which a new model has been developed based upon a previous one (Martínez-Reina et al., 2021) to include the level of microstructural damage as a new parameter regulating mechanobiological feedback. The also new proliferation function (see Figure 3) was calibrated to reproduce the Mechanostat Theory and the Principle of Cellular Accomodation. Once the new model was calibrated, it was used to simulate the response of bone to PMO, modelled as a gradual increase in RANKL expression, and subsequent treatments with denosumab. As discussed in a previous work (Martínez-Reina and Pivonka, 2019), bone density gain in denosumab treatments is mainly explained by the bone mineralisation process, which makes the mineral content to reach abnormally high values once bone turnover is blocked by the drug. But such a high mineral content jeopardises bone integrity as it makes bone matrix more brittle. Moreover, microstructural damage begins to accumulate and remains unrepaired due to the suppression or decrease of bone resorption. Figure 5 (bottom) showed how mineral content and damage rise simultaneously after the treatment commences, which is probably due to the concurrence of both factors: increased brittleness and unrepaired damage. In fact, damage is greater in the treatment group than in the non-treatment one for a long time, despite the greater BMD reached with the treatment, which increases stiffness and reduces strains. In the long-term, this reduction of strains predominates over the two negative factors commented before and damage falls below the values of the non-treatment group.

The risk of suffering an atypical femoral fracture (AFF) might also be enhanced by other factors not considered in this work. For example, at the micro (local) level, it could be affected by alterations of bone microarchitecture or by the higher concentrations of advanced glycation end-products within the extracellular collagen matrix, which can also raise bone brittleness (Vashishth et al., 2001). At the macro (organ) level, AFF might be affected by a redistribution of loads, mechanical properties, density, damage and mineral content. For example, the increased thickness of the femoral cortex observed in patients who have suffered an AFF (Larsen and Schmal, 2018) has been associated to an augmented strength and stiffness of the bone, which makes the skeletal structure more brittle at the same time (Donnelly et al., 2012). The consideration of these factors requires the use of FE models in combination with PK-PD models, as done by Hambli et al. (2016). In the present work, the risk of failure was only estimated at the local level through *NDtF*, but this limitation could be overcome by applying the present algorithm to such FE models.

### 4.2. Disease and Treatment 60Q6

The simulation of the non-treatment case predicted that the onset of the disease triggers the risk of local failure after a couple of years (see black line in Figure 5 top right). This occurs because damage is initially reduced by an increasing bone turnover rate (bone resorption is able to repair damage quite efficiently) and this decrease in damage compensates for the decrease in bone density, making *NDtF* to be approximately constant. In the mid-term, density has fallen so much that bone stiffness is significantly diminished. Strain increases and consequently damage rises, with bone resorption unable to repair the large amount of damage. The concurrence of damage increase and density decrease makes *NDtF* fall abruptly (higher risk of failure). In the long-term, though there is still an increasing risk of failure, *NDtF* reaches a stable value.

Anti-resorptive treatments lead to a decrease of bone turnover rate entailing an increase of mineral content and a subsequent increase of damage (see red line in Figure 5 bottom left), which is initially even higher than in the non-treatment group. However, in the long-term, bone density gain counteracts this effect by increasing stiffness and reducing strains, making damage slow down and, eventually, reducing the risk of failure.

### 4.3. Time Elapsed From the Onset of Disease to the Beginning of the Treatment

One factor that could limit the benefits of the treatment is the delay in its commencement. As stated before, non-treated patients could suffer a rapid bone loss right after the onset of disease, which would get stabilised after 10–12 years. However, damage did not stop rising in our simulations (though it did quite slowly in the long-term) and this made the risk of failure increase a little. This result highlights the importance of starting the treatment as soon as possible, before damage goes up excessively. Figure 6 analysed the effect of the time elapsed from the onset of disease to the beginning of the treatment. It could be seen how a late treatment can have severe consequences, as bone density gain is lower than in early treatments and, more importantly, because damage may rise even above the values reached in non-treated patients. This damage increase is quite remarkable in late treatments and is probably due to the deterioration of fatigue properties, the decrease of damage repairing and an insufficient increase of bone density and stiffness. *NDtF* shows how the combination of all these effects increased the risk of failure in late treatments. Although all of the *NDtF* curves seemed to converge to the same evolution, the period right after the start of the treatment was significantly more dangerous in late treatments.

### 4.4. Combination of Treatment and Exercise

The influence of exercise in combination with the denosumab treatment was analysed to conclude that, in general, an increment of the applied stress coincident with the commencing of the treatment is beneficial since it produces a greater bone density gain while keeping damage under control. The simulations showed that an increment of exercise is followed by a decrease of the risk of failure. On the contrary, a decrease of mechanical loading reduces the effectiveness of the treatment and this could seriously compromise bone integrity [see case 3 (dark blue line), in Figure 7]. In this case, bone formation is not promoted by the low stress and cannot compensate the bone density fall at the end of every treatment cycle, when bone turnover is slightly reactivated.

Case 5 (cyan in Figure 7) involved a strong increase of the stress in a late treatment and was analysed to illustrate three important ideas: (1) how dangerous late treatments can be; (2) the enhanced risk of failure that a significant increase of stress might produce and (3) how apparently small values of *d* ~ 0.05 are indeed high and can easily lead to failure in a few number of cycles. Late treatments were shown in Figure 6 to enhance damage accumulation and produce a limited bone density gain. This effect, in combination with a strong increment of stress, that also contributes to increase damage, led to a fatigue failure (*d* = 1 and *NDtF* = 0) soon after the beginning of the treatment.

It must be noted that all the cases analysed in Figure 7 implied overloads resulting in normal/moderate constant stresses. No high overloads or traumatic events were considered, which could cause significantly higher stresses, eventually leading to high-energy fractures. These high overloads were indeed considered in the calculation of *NDtF*, which provided the remaining fatigue life if the stress was increased up to 24 MPa, i.e., in case of an extra overload. In this regard, it is interesting to note that case 3 (underload by 10%) presented the highest risk of failure up to day 5,000 and yet case 5 was the only one to undergo failure. This is explained by the different performance of bone in both situations. In case 3, the risk of failure was determined by a low stiffness and seemed to be not so sensitive to overloads. Meanwhile, in case 5 it was determined by the high amount of unrepaired damage, which made bone more prone to failure in the event of a sudden overload, at least in the local scale, at the RVE. Certainly, the situation could be different at the organ level, as the reduction of stiffness of case 3 could redistribute the loads within the bone, what could initiate fracture elsewhere in the same bone.

The choice of 24 MPa as the overload to calculate *NDtF* was arbitrary, but allowed an easier comparison of the presented results and a more comprehensive analysis of the different factors that affect the risk of failure. The results were qualitatively similar for overloads around 24 MPa. For higher overloads, *NDtF* was very small regardless of the case, while lower overloads did not lead to a significant increase of the risk of failure in any case.

### 4.5. Importance of Brittleness vs. Unrepaired Damage in the Risk of Failure

Decrease in bone turnover due to anti-catabolic treatments has been associated to the development of AFF (Saita et al., 2015), due to the alteration of the tissue repair process (Mashiba et al., 2000). The accumulation of unrepaired microstructural damage results in unimpeded crack progression and may eventually lead to that type of fracture (Ettinger et al., 2013). However, another factor could also contribute to that increased occurrence of AFF, as hypothesised in a recent work (Martínez-Reina et al., 2021). This factor is bone mineral content, which increases as a consequence of the suppression of bone turnover, i.e., while mineral is not prevented from being accumulated within bone matrix and is not returned to blood serum by bone resorption. The mineral phase makes bone matrix more brittle, increasing the stiffness but reducing the fracture toughness of bone (Bala et al., 2013). This fact was confirmed by experimental studies that measured a higher amount of microstructural damage in interstitial bone, which has a higher BMC (Boyce et al., 1998; O'Brien et al., 2000; Qiu et al., 2005) and it was modelled in subsection 2.5 through the degradation of bone fatigue properties with the mineral content. In the above-mentioned work (Martínez-Reina et al., 2021), the risk of failure was not assessed and it was only associated to a high BMC. The present model was intended to evaluate the risk of failure by adding the damage level to the previous model as a new variable and considering damage accumulation by fatigue and damage repair by resorption. The new hypothesis is that it must be the concurrence of both factors (accumulation of unrepaired damage and increased brittleness) what would explain the high risk of AFF in antiresorptive treatments. The comparison made in Figure 6 aimed at discerning the relevance of those factors on the risk of AFF. From those simulations it could be concluded that both would play a role in the occurrence of AFF. If the stress is increased by 30%, the degradation of fatigue properties plays a key role in the occurrence of failure, as it only occurs if those properties are degraded. However, if the stress is increased by 40%, the accumulation of unrepaired damage is more important, as it is so fast that failure would occur regardless of whether the fatigue properties are degraded or not.

### 4.6. Limitations of the Study and Final Comments

The main limitation of this study is that the model was applied at the RVE level and the equations were solved only at this local level, without any interaction with the surroundings. Apart from diffusive terms of cell populations and biochemical factors, the distribution of stresses would play a key role in the behaviour at the organ level. Local changes in porosity, mineral content and damage would produce local changes in stiffness that might redistribute loads and affect the organ globally. To consider this, the model could be implemented in a Finite Element (FE) code. This would allow to evaluate that redistribution, which is particularly important in damage propagation and consequently in the assessment of the risk of fracture at the organ level. Moreover, the FE model would allow to simulate stress states more general than the simplistic uniaxial state modelled here. Nonetheless, this is out of the scope of the present paper and is left for future studies.

The risk of failure was also evaluated in a simplistic way: by estimating the remaining fatigue life of the RVE at a given time point in case of a constant overload and if all the variables except damage remained constant until failure. This estimated risk of failure should be considered only for the purpose of qualitative comparisons, as done here, and making a clear distinction between the risk of local failure, assessed at the RVE, and the risk of fracture, assessed at the organ level. As stated above, damage propagation and redistribution of stresses at the organ level would have a fundamental influence on the true risk of fracture of a given bone, but other factors such as microstructure or the multiaxiality of loads, not considered here, would also have it.

In our mineralisation algorithm, calcium (and phosphorus) availability is unlimited for deposition in bone matrix. This is only a simplification, as its availability depends on calcium (and phosphorus) homeostasis at the body level. Peterson and Riggs (2012) developed a model that accounted for calcium balance in blood serum. This process is controlled mainly by three mechanisms: (1) absorption in the intestine, (2) filtration/recirculation in the kidneys, and (3) deposition or retrieval from bone matrix through bone resorption. That balance is affected by numerous factors that could reduce mineral availability for bone deposition, among which there could be dietary restrictions and pathologies such as hypoparathyroidism or renal dysfunction. The incorporation of these mechanisms into the present model is currently under development.

Clinical results of bone density gain in denosumab treatments for PMO (Miller et al., 2008) exhibit a great variability that could be explained by the high number of factors that affect bone response. The present model is based on a previous one (Martínez-Reina et al., 2021) that was validated with the mentioned clinical results. Though not shown here, the algorithmic novelties incorporated into the present model did not produce significant changes in the model predictions and, particularly, the predicted bone density gain remained within the commented variability. Among the factors that may explain this variability we have investigated two of them: time elapsed from the onset of the disease and level of physical activity, though other factors such as treatment dose and frequency, incidence of the disease, patient's age and body weight, etc. could also have a great impact on the evolution of bone density gain and should be analysed in future studies.

Another aspect that this variability suggests is the need for a patient specific treatment, provided that this variability comes from patients' features that can be identified and measured. Obviously, this specific treatment should not conflict with WHO recommendations and therefore, they could not include dosage and frequency as variables, because these are fixed to 60Q6 for the treatment of PMO; however, other variables such as the activity level certainly could. For example, a gradual increase in activity level could be prescribed to old women who commence the treatment late. The parameters of the training regimen could be optimised as a function of patient's age, body weight, recent DEXA scans, etc. For this reason, it is of paramount importance to analyse the influence of more factors in order to design patient specific anti-resorptive treatments, which is the global aim of the present study.

## Data Availability Statement

The original contributions presented in the study are included in the article/supplementary material, further inquiries can be directed to the corresponding author/s.

## Author Contributions

JM-R and PP conceived, planned the computational simulations, and wrote the manuscript. JC-G and JM-R carried out the computational simulations. All authors discussed the results, reviewed, and commented on the manuscript.

## Conflict of Interest

The authors declare that the research was conducted in the absence of any commercial or financial relationships that could be construed as a potential conflict of interest.

## Figures and Tables

**Figure 9:**
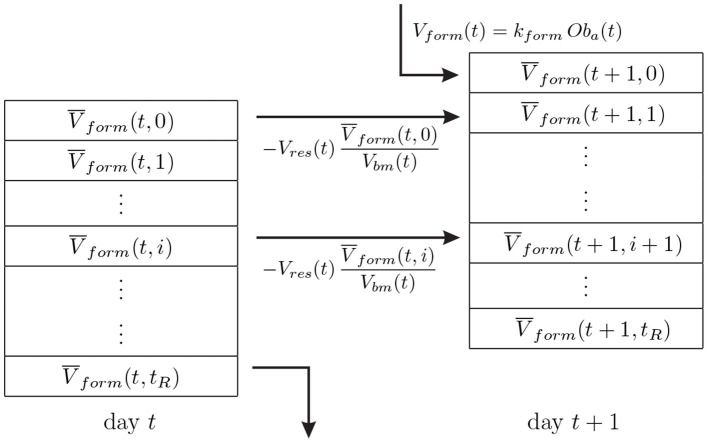
FIFO (first in - first out) queue algorithm used to update the distribution of tissue patches of different ages within the RVE.

**Figure 10:**
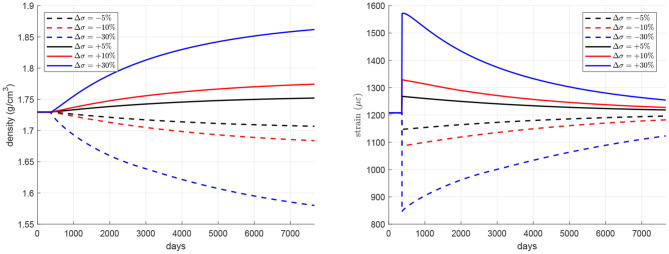
Variation of density and strain under uniaxial loading simulating underuse and overuse states.

## Footnotes

^1^ζ = 1 represents unrestricted access to denosumab, whereas ζ < 1 reflects restricted access, for example due to bone marrow being present or low blood perfusion. Hence, the denosumab concentration is bone site specific.

^2^Typical values of variable *d* are around 0.01. This makes $\Pi_{act}^{dam}\sim 10^{-3}$, while in Equation 7 $\Pi_{act}^{PTH}\sim 10^{-2}$ (see Pivonka et al., 2008). With these values, the contributions of both terms in Equation (7) are similar given that the population of osteocytes (*Ot*) is typically one order of magnitude greater than that of osteoblasts precursor cells (*Ob*_*p*_).

^3^In the isotropic damage theory, Equation (14) can be similarly written in terms of the respective Young's moduli, *E* and *E*_0_, as *E* = (1 − *d*)*E*_0_ (Pattin et al., 1996; Zioupos, 1998). Subsequently, we will use the latter formulation.

^4^In practical terms this condition was replaced by *d*(*t* + *NDtF*) = 0.999 to avoid the negative stiffness that could arise from *d* > 1 (see Equation 26).

^5^We will identify the onset of the disease with the instant when the increase in RANKL production starts, though the disease is likely not evident at that point.

^6^It must be recalled that *NDtF* is inversely related to the risk of failure: the fewer days are needed to reach d = 1, the higher is the risk of failure.

^7^These changes of load must not be confused with the overload considered in the assessment of *NDtF*. For example, in case 3 the load does change from the homeostatic value to 90% of that value at the moment the treatment begins. Then, *NDtF*(σ, *t*) is calculated for the hypothetical case that the load were increased to σ.

## Appendix

### Algorithm of Bone Mineralisation

Bone tissue is made up of bone matrix and pores. Thus, the representative volume element, *V*_*RVE*_, can be divided into the bone matrix volume, *V*_*bm*_, and the volume of vascular pores, *V*_*vas*_. In turn, bone matrix volume is divided into inorganic (mineral), organic (mainly collagen), and water phases, respectively designated as *V*_*m*_, *V*_*o*_, and *V*_*w*_:

(29) $$\begin{array}{l} V_{RVE}=V_{bm}+V_{vas}=V_{m}+V_{o}+V_{w}+V_{vas} \end{array}$$

The volume fractions of extravascular bone matrix and vascular pores are respectively given by *f*_*bm*_ = *V*_*bm*_/*V*_*RVE*_ and *f*_*vas*_ = 1 − *f*_*bm*_ = *V*_*vas*_/*V*_*RVE*_. The mineral content is usually measured by the so-called ash fraction, the ratio between mass of mineral *m*_*m*_ (or ash mass) and dry mass (the sum of inorganic and organic mass):

(30) $$\begin{array}{l} \alpha =\frac{m_{m}}{m_{m}+m_{o}}=\frac{\rho_{m}V_{m}}{\rho_{m}V_{m}+\rho_{o}V_{o}} \end{array}$$

where density of hydroxyapatite is taken for $\rho_{m}=3.2g/cm^{3}$ (Currey, 2004). Organic phase is mainly composed of type I collagen, but other non-collagenous proteins are also present. Thus, $\rho_{o}=1.2g/cm^{3}$ was adjusted to provide a tissue density $\rho_{t}=2.1g/cm^{3}$ for the completely mineralised tissue α = 0.73 (García-Aznar et al., 2005).

Specific volumes are defined by: *v*_*o*_ = *V*_*o*_/*V*_*bm*_, *v*_*m*_ = *V*_*m*_/*V*_*bm*_, and *v*_*w*_ = *V*_*w*_/*V*_*bm*_, which implies that *v*_*o*_ + *v*_*w*_ + *v*_*m*_ = 1. Then, Equation (30) can be given in terms of the specific volumes:

(31) $$\begin{array}{l} \alpha =\frac{\rho_{m}v_{m}}{\rho_{m}v_{m}+\rho_{o}v_{o}} \end{array}$$

Bone tissue density is given by:

(32) $$\begin{array}{l} \rho_{t}=\frac{m}{V_{bm}}=\rho_{m}v_{m}+\rho_{o}v_{o}+\rho_{w}v_{w} \end{array}$$

Mineral accumulates by displacing water present in bone matrix (Hernandez et al., 2001a). Therefore, the volume ratio of organic phase is assumed constant during the mineralisation process, *v*_*o*_ = 3/7 (Martin, 1984); while the variations of mineral and water volume ratios hold Δ*v*_*m*_ = −Δ*v*_*w*_. We have followed Hernandez et al. (2001a) to assess the increase of *v*_*m*_ with time, by distinguishing the mineralisation lag time; the primary phase, with a linear increase, and the secondary phase, with an exponentially decreasing rate:

(33) $$\begin{array}{l} v_{m}\left(t\right)=\{\begin{array}{ll} 0 & \text{if }t\leq t_{mlt}\\ v_{mprim}\frac{t-t_{mlt}}{t_{prim}} & \text{if }t_{mlt}<t\leq t_{prim}+t_{mlt}\\ v_{mmax}-\left(v_{mmax}-v_{mprim}\right)\\ e^{-\kappa \cdot \left(t-t_{prim}-t_{mlt}\right)} & \text{if }t_{prim}+t_{mlt}<t \end{array} \end{array}$$

where *t*_*mlt*_ and *t*_*prim*_ are, respectively, the length of the mineralisation lag time and the primary phase; *v*_*m prim*_ is the mineral specific volume at the end of the primary phase, corresponding to α = 0.45 (Hernandez et al., 2001a); *v*_*m max*_ is the mineral specific volume corresponding to the maximum calcium content, 300 *mg*/*g* (Currey, 2004); and κ is a parameter measuring the rate of mineral deposition during the secondary phase (see Table A1). Note that, with the assumptions made above, Equations (31) and (32) establish a biunivocal relation between α and ρ_*t*_. Also, according to Equation (25), apparent density is univocally determined by *f*_*bm*_ and α.

**Table A1:** Values taken for the constants of the PK-PD model.

**Parameter**	**Value**	**Units**
*k*_*res*_	2	day^−1^
*k*_*form*_	0.4	day^−1^
*Ob*_*u*_	0.01	pM
*Oc*_*p*_	0.001	pM
*D*_*Ob*_*u*__	6.3 · 10^−4^	day^−1^
*D*_*Ob*_*p*__	1.657 · 10^−1^	day^−1^
*P*_*Ob*_*p*__	0.211	day^−1^
*D*_*Oc*_*p*__	2.1	day^−1^
*A*_*Ob*_*a*__	0.211	day^−1^
*A*_*Oc*_*a*__	5.65	day^−1^
β_*RANKL*_	168.4	pM day^−1^
${\tilde{D}}_{RANKL}$	10.13	day^−1^
*R*_*RANKL*_	2.7 · 10^6^	-
*R*_*RANK*_	1 · 10^4^	-
β_*OPG*_	1.625 · 10^8^	pM day^−1^
${\tilde{D}}_{OPG}$	0.35	day^−1^
*OPG*_*max*_	2 · 10^8^	pM
ζ	0.02	-
*K*_*d*, [*RANKL*−*OPG*]_	1000	pM
*K*_*d*, [*RANKL*−*RANK*]_	29,31	pM
*K*_*d*, [*RANKL*−*den*]_	3	pM
η	4.143 · 10^−2^	pM
σ	12	MPa
$P_{RANKL}^{mech,max}$	500	pM day^−1^
$P_{RANKL}^{PMO,max}$	600	pM day^−1^
δ_*PMO*_	2	years
${\tilde{D}}_{TGF-\beta }$	2	day^−1^
α_*TGF*−β_	1	-
$K_{act}^{TGF-\beta }$	5.633 · 10^−4^	pM
$K_{rep}^{TGF-\beta }$	1.754 · 10^−4^	pM
$K_{act}^{PTH}$	150	pM
$K_{rep}^{PTH}$	0.223	pM
*k*_*a*_	0.17	day^−1^
*k*_*el*_	1.15 · 10^−2^	day^−1^
*V*_*c*_	779	ml kg^−1^
*F*	1	-
*K*_*m*_	411	ng ml^−1^
*V*_*max*_	2672	ng kg^−1^ day^−1^
BW	60	kg
*t*_*mlt*_	12	days
*t*_*prim*_	10	days
*v*_*mprim*_	0.121	-
*v*_*mmax*_	0.442	-
κ	0.005	-
*t*_*R*_	3000	days

The amount of mineral contained in a RVE depends on the age of the tissue through Equation (33), but the RVE can be made up of tissue patches formed in the recent history, viz. of different ages. Moreover, the tissue within the RVE can be resorbed, which puts the mineral back into the blood flow. The amounts of tissue of different ages contained in the RVE are estimated using the algorithm depicted in Figure 9 (Martínez-Reina et al., 2009). ${\bar{V}}_{form}\left(t,\tau \right)$ provides the bone volume formed τ days ago and still present (not yet resorbed) at time *t*. Knowing the distribution of tissue patches of different ages at day *t* (left column) and the volume formed (*V*_*form*_(*t*) = *k*_*form*_*Ob*_*a*_(*t*)) and resorbed that day (*V*_*res*_(*t*) = *k*_*res*_*Oc*_*a*_(*t*)), the distribution at day *t* + 1 (right column) can be estimated:

(34) $$\begin{array}{l} {\bar{V}}_{form}\left(t+1,i+1\right)={\bar{V}}_{form}\left(t,i\right)-V_{res}\left(t\right)\frac{{\bar{V}}_{form}\left(t,i\right)}{V_{bm}\left(t\right)} \end{array}$$

Finally, the mineral content of each patch is summed to estimate the average mineral content of the RVE at day *t* + 1.

(35) $$\begin{array}{l} v_{m}\left(t+1\right)=\frac{\sum_{i=0}^{t_{R}}{\bar{V}}_{form}\left(t+1,i\right)\cdot v_{m}\left(i\right)}{V_{bm}\left(t+1\right)} \end{array}$$

where the mineral contents of the patches, *v*_*m*_(*i*), are calculated through Equation (33). *t*_*R*_ represents the residence time, e.g., the typical time the patch tissue remains within the bone before being resorbed. This residence time can be very large but the queue can be truncated at a shorter time to reduce the computational cost. See (Martínez-Reina and Pivonka, 2019) for more details.

It must be noted here that the recursive character of this mineralisation algorithm makes it necessary to integrate the set of differential equations that governs the model using an explicit integration scheme.

### Adjustment of Mechanoregulation

The points that delimit the piecewise linear function $\Pi_{act}^{\psi_{bm}}$ in Figure 3 were adjusted to accomplish the Mechanostat Theory and the Principle of Cellular Accommodation. The Mechanostat Theory establishes and “adapted window” between 800 and 1,200 με (Frost, 2003), as were the homeostatic strains obtained in Figure 4.

The Principle of Cellular Accommodation establishes that bone cells react strongly to variations in their environment, but eventually “accommodate” to steady state signals (Turner, 1999), in such a way that those variations in the stress would produce a transient response to adapt to the new environment. To check if the proposed model accomplished with that principle, a set of simulations were run starting from the homeostatic situation and implementing variations of the stress that were kept constant to let bone reach a new steady state (see Figure 10). Increments of stress lead to an increase in bone density and stiffness (reversely in the case of decrements of stress), while strains lie out of the “adapted window” defined by the Mechanostat Theory. Bone tends to return to that strain range and accommodates to the new environment when the “adapted window” is reached. The limits established in Figure 3 to define $\Pi_{act}^{\psi_{bm}}$ were adjusted to achieve the behaviour of Figure 10, with an equilibrium strain around 1,200 με, as stated before, and a slope for the adaptation response small enough not to produce numerical instabilities and large enough to achieve a new equilibrium in a reasonable time (70% of the total change reached after 8–10 years).

### One Compartment PK Model of Denosumab

Among the pharmacokinetic (PK) models of denosumab that can be found in the literature, we have followed the one proposed by Marathe et al. (2011). This is a one-compartment model with Michaelis-Menten kinetics that estimates serum denosumab profiles. This model includes a first-order rate process (constant *k*_*a*_) governing the absorption of the drug (input parameter *Dose*_*den*_) from the subcutaneous injection site into the central compartment (variable *C*_*P, den*_ and constant *V*_*c*_). Drug elimination from the central compartment is described by a combination of a linear first-order process (constant *k*_*el*_) and a non-linear saturation process (constants *V*_*max*_, *K*_*m*_):

(36) $$\begin{array}{l} \frac{dC_{p,den}}{dt}=k_{a}\frac{Dose_{den}}{V_{c}/F}\cdot e^{-k_{a}t}-\frac{C_{p,den}}{K_{m}+C_{p,den}}\frac{V_{max}}{V_{c}/F}-k_{el}\cdot C_{p,den} \end{array}$$

where, *V*_*c*_ is the volume of the central compartment and the factor *F* is the bioavailability, which is equal to 1 when the drug is administered intravenously, as assumed here. In Equation (36) *Dose*_*den*_ is given in ng of denosumab per kg of body weight^8^. *C*_*p, den*_ is the concentration of denosumab in the central compartment, calculated (in ng/ml) as a function of time by solving the differential equation (36) and subsequently converted into pmol/l, through the molecular weight of denosumab *M*_*den*_ = 149 kDa (Amgen). The initial condition for Equation (36) is set to zero, indicating the absence of drug. The prolonged absorption phase and the absence of intravenous data precludes the need for including distribution of the drug to a non-specific tissue compartment and thus reduces the number of parameters in this model.

### Biochemical Regulatory Factors

TGF–β is stored in the bone matrix and released during resorption by osteoclasts. Its concentration is calculated following Pivonka et al. (2008):

(37) $$\begin{array}{l} TGF-\beta =\frac{\alpha_{TGF-\beta }k_{res}Oc_{a}}{{\tilde{D}}_{TGF-\beta }} \end{array}$$

where α_*TGF*−β_ is the concentration of TGF–β in bone matrix and ${\tilde{D}}_{TGF-\beta }$ is the TGF–β degradation rate. The concentration of TGF–β is used to define the activator/repressor functions in Equations (1)–(3):

(38) $$\begin{array}{l} \Pi_{act}^{TGF-\beta }=\frac{TGF-\beta }{K_{act}^{TGF-\beta }+TGF-\beta } \end{array}$$

(39) $$\begin{array}{l} \Pi_{rep}^{TGF-\beta }=\frac{K_{rep}^{TGF-\beta }}{K_{rep}^{TGF-\beta }+TGF-\beta } \end{array}$$

with $K_{act}^{TGF-\beta }$ and $K_{rep}^{TGF-\beta }$ the activation and repression constants, respectively. RANK is expressed by osteoclasts precursors:

(40) $$\begin{array}{l} RANK=R_{RANK}\cdot Oc_{p} \end{array}$$

where *R*_*RANK*_ is the carrying capacity. Concentration of OPG is downregulated by PTH and is calculated following Pivonka et al. (2012):

(41) $$\begin{array}{l} OPG=\frac{\beta_{OPG}Ob_{a}\Pi_{rep}^{PTH}}{{\tilde{D}}_{OPG}+\frac{\beta_{OPG}Ob_{a}\Pi_{rep}^{PTH}}{OPG_{max}}} \end{array}$$

where β_*OPG*_ and ${\tilde{D}}_{OPG}$ are, respectively, the production and degradation rates of OPG and the activator/repressor functions that govern PTH regulation on the RANKL-RANK-OPG signaling pathway (Equations 41, 7) are given by:

(42) $$\begin{array}{l} \Pi_{act}^{PTH}=\frac{PTH}{K_{act}^{PTH}+PTH} \end{array}$$

(43) $$\begin{array}{l} \Pi_{rep}^{PTH}=\frac{K_{rep}^{PTH}}{K_{rep}^{PTH}+PTH} \end{array}$$

where the concentration *PTH* = 2.91 pM has been assumed constant in this case (Pivonka et al., 2008) and $K_{act}^{PTH}$ and $K_{rep}^{PTH}$ are the corresponding activation and repression constants.

The values of the constants of the PK-PD model are given in Table A1. A detailed discussion of these values can be consulted in (Martínez-Reina and Pivonka, 2019; Marathe et al., 2011; Scheiner et al., 2014).
